# College students’ knowledge, attitudes, and practices of garbage sorting and their associations: a cross-sectional study of several universities in Beijing, China

**DOI:** 10.3389/fpubh.2024.1328583

**Published:** 2024-02-16

**Authors:** Siyuan Liu, Xiong Liu, Yibo Li, Dongli Yang, Feng Li, Junling Yang

**Affiliations:** School of Marxism, China University of Geosciences, Beijing, China

**Keywords:** garbage sorting, knowledge, attitude, behavior, college students

## Abstract

**Background:**

In recent years, the Chinese government has placed growing emphasis on environmental development. The implementation of effective waste separation practices in schools is crucial for establishing an ecological civilization in China.

**Objective:**

The present study aimed to assess the knowledge, attitude, and practice (KAP) of waste separation among Chinese university students and to understand the demographic factors influencing the KAP of the interviewed students. These sociodemographic factors include gender, age, education, and family environment.

**Methods:**

Based on the KAP theoretical model and the Lewin behavioral model (LBM), this study developed its questionnaire on college students’ KAP of rubbish sorting. A survey was conducted on 1,282 college students from five colleges and universities in Beijing. A one-way ANOVA, Pearson’s correlation analysis, and multiple linear stepwise regression analyzes were employed to explore the factors influencing college students’ KAP scores on waste sorting. The questionnaire’s reliability and validity were effectively verified through two rounds of Delphi expert consultation.

**Results:**

The scores for KAP dimensions were 55.64, 69.18, and 54.8%, respectively. The overall KAP score of university students in waste classification was 46.93 ± 9.93, with a percentage score of 62.57%. More than half of the college students lack a clear understanding of waste classification. Grade, gender, major, highest family education, and family economic status all influence college students’ KAP scores on waste classification. There is a notable deficiency in school education regarding waste classification, with only 30.7% reporting having received such education.

**Conclusion:**

This study unveils the overall KAP score of waste separation among Chinese college students, which is marginally acceptable. The interviewed students exhibit a positive attitude and a willingness to participate in waste separation. However, there is room for improvement in both knowledge and practices. A lack of knowledge about waste sorting emerges as the primary influence on individual-level practices. Consideration should be given to enhancing education and management of waste separation among college students, emphasizing the cultivation of an eco-conscious culture, and guiding students to establish correct ecological values.

## 1 Introduction

With the continuous advancement of urbanization, the phenomenon of “garbage-enclosed cities” has become an increasingly serious environmental problem in the current times, garnering attention and recognition across all sectors of society (1). In the 2020 Annual Report on Prevention and Control of Solid Waste Pollution in Large and Medium-Sized Cities in China published by China’s Ministry of Ecology and Environment, it is mentioned that the amount of domestic waste generated in the country’s 196 large and medium-sized cities in 2019 reached a staggering 235.602 million tons (2). According to the China Urban Environmental Sanitation Association, China generates nearly 1 billion tons of rubbish annually, with a growth rate of 5–8% per year (3). In most areas of China, domestic waste is still disposed of in mixed landfills and incinerators, causing significant secondary pollution to water, soil, and air. This practice disrupts the balance between the economy, society, and the ecosystem (4). Large quantities of unsegregated domestic waste has resulted in high disposal costs in cities, imposing a significant burden on the ecosystem. In December 2016, General Secretary Xi Jinping proposed the implementation of the segregation of domestic waste and provided important instructions regarding the work of urban and rural domestic waste segregation (5). In the subsequent years, various departments in China issued several policy documents, emphasizing the necessity of comprehensively implementing on-site classification and source reduction treatment of domestic waste (6–8). The objective is to promote the economical and intensive use of all types of resources and expedite the construction of a waste recycling system (9). In recent times, colleges and universities, identified as the “main battlefield” and a “key focus area” for waste classification, have also garnered attention of the Chinese government. At the forefront of waste classification initiatives, cities such as Beijing, Shanghai, and Guangzhou have successfully revised and implemented regulations on residential waste management. Additionally, educational authorities worldwide are aligning with national policy requirements, issuing documents such as the “Work Program for Classification of Living Garbage in Schools,” “Guidelines for Classification of Living Garbage in Schools (for Trial Implementation),” and “Supervision of Garbage Classification in the Educational System.” These measures aim to incorporate waste classification as a crucial component, considering it a fundamental aspect within the broader context of education. The emphasis on the “key small matter” of garbage classification is integrated into the overall educational landscape and is being comprehensively planned. Simultaneously, the documents also highlight that college students, being the most active and energetic members of the youth group, should serve as leaders and practitioners in waste classification. Furthermore, in terms of the waste classification process, the source classification and disposal of waste by college students constitute the foundational step in the entire classification system. This action is crucial for the middle-end classification collection, classification transportation, and the end classification and disposal processes (10). Thus, the source classification and disposal of waste by college students is directly connected to the progress of subsequent sessions, as well as the cost and effectiveness of sorting and processing on campus.

However, previous studies have found that waste segregation on campus is unsatisfactory, and the results of different surveys are mixed (11–13). The main reasons for this phenomenon can generally be attributed to four aspects (14). First, the number of students in China is increasing year by year, resulting in a corresponding increase in the amount of waste generated. Additionally, there are many types of waste inside university campuses, leading to an increasingly serious situation of waste classification (15) Second, the current education on waste classification in some Chinese universities is ineffective, and students lack the knowledge of waste classification, creating obstacles to the practical implementation of waste classification (16). Third, the supervision of waste classification in Chinese universities is inadequate and is influenced by individual behavior. The uncertainty of time and space, combined with the low economic benefits of waste separation and disposal for participants and the lack of appeal, significantly increases the difficulty of waste separation supervision (17). Fourth, students’ ingrained lifestyle habits pose a challenge, as some students find waste classification and disposal too troublesome (18).

Previous studies have indirectly suggested variations in the ability of university students to separate rubbish. A systematic evaluation showed that college students’ waste classification behavior is influenced by the quality of ecological civilization education in colleges and universities. College students with substantial knowledge reserves related to resource conservation, ecological and environmental protection, and waste classification exhibited better performance in terms of waste classification practices (19). This suggests that there is a need to enhance college students’ ability related to waste classification, as indicated by Zhang’s study, which shows that the overall cognitive average of college students’ waste classification behavior is only 3.69 out of 5 (20). There is a significant correlation between college students’ knowledge base of waste classification and their performance in waste classification (21). Hence, it is imperative for college students to possess the knowledge and skills of waste classification. They need to acquire the understanding, emotional recognition, and practical implementation of waste classification in their daily lives to ameliorate the unsatisfactory state of waste classification in colleges and universities. The knowledge, attitude, and practice (KAP) of university students play a crucial role in determining the success of waste separation in universities.

However, few studies have directly explored the KAP status of university students’ waste sorting, and the level of KAP related to waste sorting among university students remains unclear. The current research by Chinese scholars on this kind of problem often focuses on the waste classification status of urban or rural residents. They analyze the challenges these residents face and explore potential countermeasures. However, very few scholars focus on the waste classification status of college students within higher education institutions. Moreover, this kind of research tends to focus on a specific type of colleges and universities, lacking overall representativeness (1, 11). In terms of research content, their research predominantly concentrates on the knowledge dimension, with limited exploration of the specific behaviors and attitudes of college students in garbage classification. In terms of research content, their studies mainly concentrate on the knowledge dimension, with few investigations into the specific performance of college students in rubbish classification within the attitude and practice dimensions (11, 22, 23). In addition, researchers in Europe, North America, and South America typically emphasize improving behavioral outcomes. They are more inclined to use specific methods and economic strategies to promote waste reduction and the sustainable use of waste (24, 25). Therefore, as there is currently no specific KAP questionnaire on waste classification tailored for college students in the academic world, this study employs a self-administered questionnaire guided by the theoretical framework of the KAP model and the Lewin behavioral model (LBM). The research aims to examine the KAP status of college students regarding waste classification and explore the factors influencing their KAP status in this regard. This study seeks to provide future guidance for Chinese universities in conducting ecological civilization education for college students. In addition, we also explore the barriers to waste separation in universities and colleges, detailing some of the challenges related to waste separation stemming from both internal and external factors.

## 2 Methodology

This study adopts a step-by-step approach outlined as follows: (1) identification of the primary research trends in recent literature (Section 1) and (2) drafting of the questionnaire, development of the sampling strategy, and execution of data collection (Sections 2.1, 2.2, 2.3, and 2.4), followed by data analysis (Section 2.5). This research methodology is recognized for combining qualitative and quantitative methods to attain a comprehensive understanding and an in-depth yet precise argument (26, 27). By employing stakeholder triangulation and gaining a comprehensive understanding of stakeholder feedback, this methodology is considered appropriate for ensuring data robustness and scientific soundness (28, 29).

### 2.1 Study design and subjects

Between September and November 2022, we carried out a cross-sectional study on the waste sorting practices among university students in 13 colleges across 5 universities in the Beijing area. The five universities are the China University of Geosciences (Beijing), the University of Science and Technology Beijing, the Beijing Forestry University, the Beijing Language and Culture University, and the China University of Mining and Technology (Beijing). Both undergraduate and postgraduate students were randomly selected from these institutions to participate in the study. In selecting the samples, we adhered to the principle of stratified random sampling. Undergraduates were chosen at a rate of 10% of the total number of students, while postgraduates were selected based on approximately 15 students from each college. This sampling method improves the predictive accuracy of the destination choice model for pedestrian destinations. The goodness-of-fit and correctness of the sampling method require the sampler to possess comprehensive knowledge of the overall units; otherwise, achieving a scientifically sound classification is challenging (30). To ensure the ethicality and compliance of the study, we engaged in thorough communication with the students. We clearly conveyed the purpose, methodology, expected benefits, and potential risks of the study while ensuring that their participation was voluntary and based on a full understanding of the research. Simultaneously, we implemented measures to protect the privacy and rights of the students, ensuring the security of their data. Ethical principles were strictly adhered to throughout the study, and informed consent was obtained from the students. Additionally, approval was sought from the school management unit.

### 2.2 Development of the KAP questionnaire on waste separation for university students

The KAP theory holds a prominent position among the most influential behavioral intervention theories according to American academics (31, 32). It is one of the most commonly used research models in the pursuit of health behavioral research, and it is primarily aimed at gathering information about what is known, believed, and accomplished about a specific topic within a given population (33). The KAP theory suggests that knowledge serves as the precursor to the formation of attitudes, and through attitudes, it influences practical behaviors (34). In this context, knowledge refers to the awareness or understanding of information, attitude denotes the positive or negative evaluation of a goal, and behavior refers to the regular activities carried out in response to different problems. Typically, research subjects alter their attitudes through the acquisition of additional knowledge, and then the changed attitude influences their behavior (35).

Following the KAP model theory, the KAP questionnaire on waste sorting for university students was developed by reviewing several guidelines (21, 36). To explore more details, we designed a KAP questionnaire containing several types of questions: single choice, multiple choice, scale (measured on a five-point Likert scale), and sorting. Each single-choice question has only one correct answer, and multiple-choice questions has multiple correct answers. The questionnaire consisted of 39 items divided into 5 sections: knowledge dimension (10 items covering the information about waste that commonly requires sorting in schools), attitude dimension (8 items, mainly measured on an 8-point scale), practice dimension (5 items, all single choice questions), and school education and management (6 items, including 5 single choice and 1 multiple choice questions). In addition, we designed two auxiliary questions related to the knowledge dimension and one auxiliary question related to the schooling and management dimension. In the design of the questionnaire content, we followed three principles: representativeness, clarity, and interpretation. This involved (1) ensuring a uniform distribution of survey respondents (37), (2) formulating questions for the questionnaire in a clear and straightforward manner (38, 39), and (3) providing reasonable options to avoid excessive room for interpretation (40).

In addition, we sought input from 10 experts at the China University of Geosciences (Beijing) through a Delphi consultation during the preparation of the initial research questionnaire. Their research fields included ideological and political education, management, psychology, education, and sociology. The experts were well versed in knowledge related to waste classification, as well as the educational and management methods of waste classification. They were asked to evaluate the rationality and importance of each item in the questionnaire content and to give their opinion on each item. The questionnaire was initially developed through two rounds of the Delphi expert communication method until a basic consensus was reached among the experts. The agreement of the 10 experts was evaluated using the mean and coefficient of variation (CV) for the importance and reasonableness ratings. The mean value reflects the importance and reasonableness of each item, while the CV reflects the dispersion of each item. A CV below 0.25 is considered acceptable (41, 42). The experts rated the importance and reasonableness of each item using a five-point Likert scale, where points 1–5 represent the level of increase in reasonableness or importance. Finally, the researcher finalized the questionnaire based on the experts’ opinions.

The knowledge dimension comprises 10 items, primarily including types of waste classification, knowledge of common waste classification in schools, policies related to waste classification in Beijing, and related management measures and strategies. A total of 10 points were allocated for the knowledge dimension, awarding 1 point for a correct answer and 0 points for an incorrect answer. Additionally, we added two questions. One question inquired about the perceived level of knowledge mastery regarding waste classification for college students themselves, while the other question sought information on the main source of knowledge about waste classification among college students. Including these two questions helps us to compare the discrepancy between college students’ actual knowledge of waste classification and their self-perceived knowledge. Simultaneously, it allows us to identify the primary channels through which knowledge of waste classification is currently disseminated among college students.

The attitude dimension focuses on university students’ ratings of the importance, responsibility, and efficacy of waste separation. Scores range from 1 to 5, representing an 8-point scale from “strongly disagree” to “strongly agree” with the program. Students were also tasked with assessing the primary motivations for waste separation through a multiple-choice question. We also investigated the willingness of university students to learn about waste separation.

In the practice dimension, a five-point Likert scale was used to explore the frequency of university students’ engagement in waste sorting practices. The scale ranged from 1 to 5, representing “never” to “always,” with a total of 5 points.

In the education and management dimension, questions were divided into two categories: education and management. In the education dimension, two single-choice questions were set to explore the provision of courses related to waste separation. These questions investigated whether the knowledge of waste separation was involved in the content of the courses, the role of school education in fostering waste separation among college students, and the reasons for triggering waste separation behaviors. In the direction of school management, three single-choice questions were set to assess the implementation status of waste separation in schools. Additionally, one multiple-choice question was included to understand the reasons behind the problem of waste separation on campus. Furthermore, we also added one question mainly to gather suggestions or opinions on waste separation in schools. This serves as a supplement to the KAP survey and is only intended to solicit suggestions for future research, such as the development of improvement measures and the enhancement of quality levels.

### 2.3 Reliability and validity of the KAP questionnaire on waste separation for university students

The reliability of the KAP questionnaire on waste sorting for university students was measured using Cronbach’s alpha coefficient and discussed within a small focus group. The internal consistency reliability test included all scoring questions. The overall Cronbach’s alpha coefficient was 0.866, which is acceptable. We also engaged six students to participate in our focus group. All focus group members were asked to provide suggestions about the structure of the questionnaire and the readability of each item. They were asked to assess whether the expression of each option was clear and whether there was any ambiguity. All members unanimously agreed that all items were easy to understand and were well structured.

Questionnaire Validity, that is, the validity and correctness of the questionnaire. It refers to the use of professional measurement tools or methods to accurately measure the effectiveness of the test object data, that is, to observe through KMO test (Kaiser-Meyer-Olkin test). Prior to conducting the research, this study conducted two pre-surveys and asked scholars in related fields to evaluate the content of the questionnaire in two rounds. They mentioned that the content of the questionnaire is closely aligned with the topic of the study, meets the characteristics and needs of the current new era development, and can effectively achieve the measurement purpose. By comprehensively analyzing the validity of the questionnaire content, we found that the cumulative contribution rate of factor variance reaches more than 50%, and the KMO value is 0.898, with the test value exceeding 0.7. These results indicate that the questionnaire has good validity, and the questionnaire survey aligns with scientific testing standards. Therefore, we state that the KAP questionnaire employed for university students’ waste classification is both reliable and valid.

### 2.4 Data collection

The data collection period spanned from September to November 2022. We printed the questionnaires and carried out the pre-survey and research process based on the predetermined number of copies. During the formal research, we distributed the questionnaires to the heads of 13 colleges in 5 universities in Beijing, adhering to the stipulated stratified sampling ratio. Subsequently, teachers from the colleges instructed the students to fill out the questionnaires. After completing the questionnaire, the respective college administrators collected and compiled them for the research team. Finally, the members of the research group manually entered the collected paper questionnaires into SPSS25.0 one by one.

### 2.5 Data analysis

SPSS 25.0 was used for both data entry and analysis. Descriptive statistics selected for each item included frequencies and percentages. Continuous variables (e.g., scores) were measured as mean (M) ± standard deviation (SD). Categorical variables were expressed in statistics as frequencies and percentages. T-tests and F-tests were used for one-way analyzes. A Pearson correlation analysis was employed to investigate the correlation between general factors, with Pearson correlation coefficient (*r*) scores being calculated. The multiple linear regression models were used to examine the factors influencing the scores. A value of p of <0.05 was considered statistically significant.

## 3 Results

### 3.1 Participant characteristics

We distributed 1,300 questionnaires and received 1,282 valid responses, resulting in a validity rate of 98.62%. Survey respondents were selected based on characteristics such as gender, major, grade, and political affiliation. Detailed information on the demographic characteristics of the sample is shown in Table 1. Through a one-way ANOVA, it was observed that various factors such as different colleges, grades, genders, majors, family economic levels, and family educational backgrounds have an impact on college students’ KAP scores on waste classification.

**Table 1:** Demographic characteristics of participants and a one-way ANOVA typology of knowledge, attitude, and practice of waste classification among university students (*n* = 1,282).

	Number (%)	Knowledge score	F	p	Attitude score	F	p	Practice score	F	p	Total score	F	p
College			8.077^b^	<0.01		5.475^b^	<0.01		3.86^b^	<0.01		2.215^b^	<0.01
School of Earth Sciences and Resources	125 (9.80%)	5.57			27.72			13.78			44.19		
School of Engineering and Technology	51 (4.00%)	5.64			27.96			13.77			45.80		
School of Materials Science and Technology	73 (5.70%)	5.67			28.01			13.77			44.60		
School of Information Engineering	131 (10.20%)	5.65			28.10			13.90			43.82		
School of Water Resources and Environment	117(9.10%)	5.81			28.46			13.95			48.36		
School of Energy Resources	91 (7.10%)	5.78			28.41			13.98			43.89		
School of Economics and Management	167 (13.00%)	5.88			28.63			14.09			47.10		
School of Foreign Languages	79 (6.20%)	5.98			28.73			14.26			49.23		
School of Gemmology	98 (7.60%)	5.97			28.66			14.23			50.59		
School of Geophysics and Information Technology	77 (6.00%)	5.90			28.42			13.93			50.61		
School of Ocean Sciences	139 (10.80%)	5.66			28.04			13.77			46.64		
School of Land Science and Technology	92(7.20%)	5.77			28.40			13.56			49.22		
School of Science	42 (3.30%)	5.76			27.73			12.99			48.81		
Grade			7.743^b^	<0.01		10.112^b^	<0.01		6.098^b^	<0.01		1.736^b^	0.001
Freshman year	286 (22.30%)	5.56			27.71			13.80			49.41		
Second grade	352 (27.50%)	5.53			27.74			13.82			47.47		
Junior class	260 (20.30%)	5.58			27.83			13.81			46.91		
Senior class	281 (21.90%)	5.61			27.89			13.79			44.35		
Graduate student	103 (8.00%)	5.61			27.22			12.64			45.47		
Gender			−4.782^a^	<0.01		−3.296^a^	<0.01		−7.521^a^	<0.01		10.469^a^	0.001
Male	734 (57.30%)	5.57			27.68			13.71			45.46		
Female	548 (42.70%)	5.56			27.69			13.71			48.93		
Political status			0.333^b^	0.802		2.177^b^	0.089		2.441^b^	0.63		1.277^b^	0.087
CPC member	203 (15.80%)	5.56			27.69			13.72			45.96		
Member of the Communist Youth League	929(72.50%)	5.56			27.68			13.71			47.27		
Democratic parties	8 (0.60%)	5.81			28.46			13.98			40.00		
Mob	142 (11.10%)	5.58			27.71			13.74			46.58		
Profession			6.378^b^	<0.01		2.767^b^	0.007		1.094	0.365		1.855	<0.01
Neo-Confucianism	374 (29.20%)	5.56			27.68			13.72			46.45		
Engineering	591 (46.10%)	5.56			27.69			13.71			47.21		
Economics	71 (5.50%)	5.58			27.78			13.77			43.63		
Management science	88 (6.90%)	5.60			27.78			13.69			45.78		
Jurisprudence	50 (3.90%)	5.93			28.83			14.30			48.14		
Literature	74 (5.80%)	5.34			27.26			13.41			49.97		
Art theory	31 (2.40%)	5.80			28.40			13.90			49.65		
Family highest education			8.802^b^	<0.01		4.17^b^	0.006		7.571^b^	<0.01		0.816^b^	0.829
Junior high school and below	290 (22.60%)	5.57			27.69			13.71			47.14		
Senior high school	402 (31.40%)	5.56			27.68			13.72			46.08		
Collegiate	496 (38.70%)	5.57			27.70			13.72			47.92		
Graduate student	94 (7.30%)	5.57			27.68			13.71			44.88		
Family economic situation			3.776^b^	0.005		6.367^b^	<0.01		7.002^b^	<0.01		1.544^b^	0.007
Very low level	85 (6.60%)	5.56			27.71			13.74			43.03		
Lower level	336 (26.20%)	5.57			27.69			13.72			45.82		
Medium level	767 (59.80%)	5.56			27.67			13.70			47.71		
Higher level	71 (5.50%)	5.58			27.78			13.80			49.48		
Extremely high level	23 (1.80%)	5.57			27.82			13.82			44.35		
Headcount	1,278	5.56 ± 2.05			27.67 ± 6.28			13.70 ± 3.97			46.94 ± 9.94		

a T-test.

b F-test.

### 3.2 University students’ knowledge and sources of knowledge about waste separation

**Figure 1:**
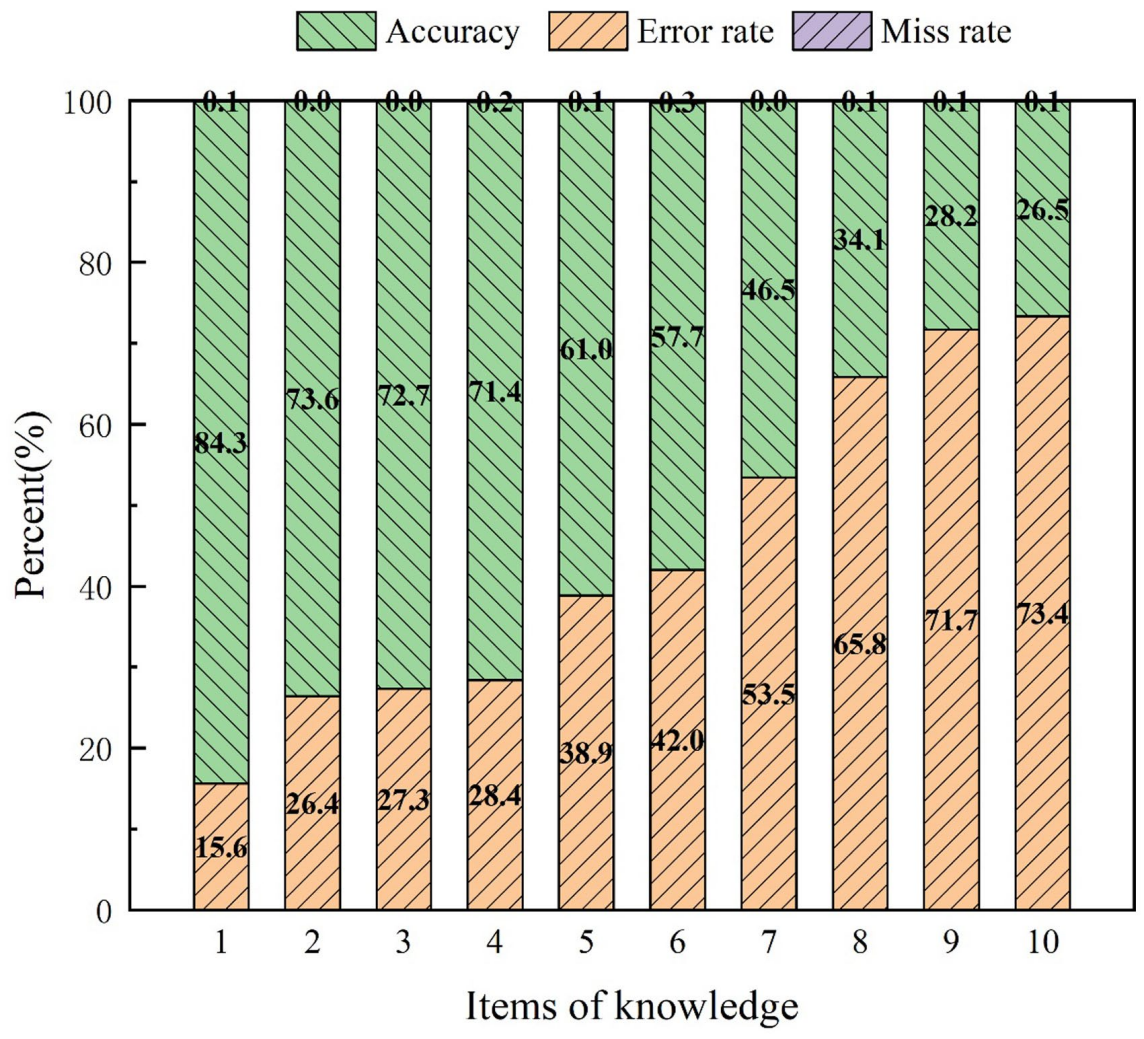
The knowledge mastery score of university students on waste classification.

**Figure 2:**
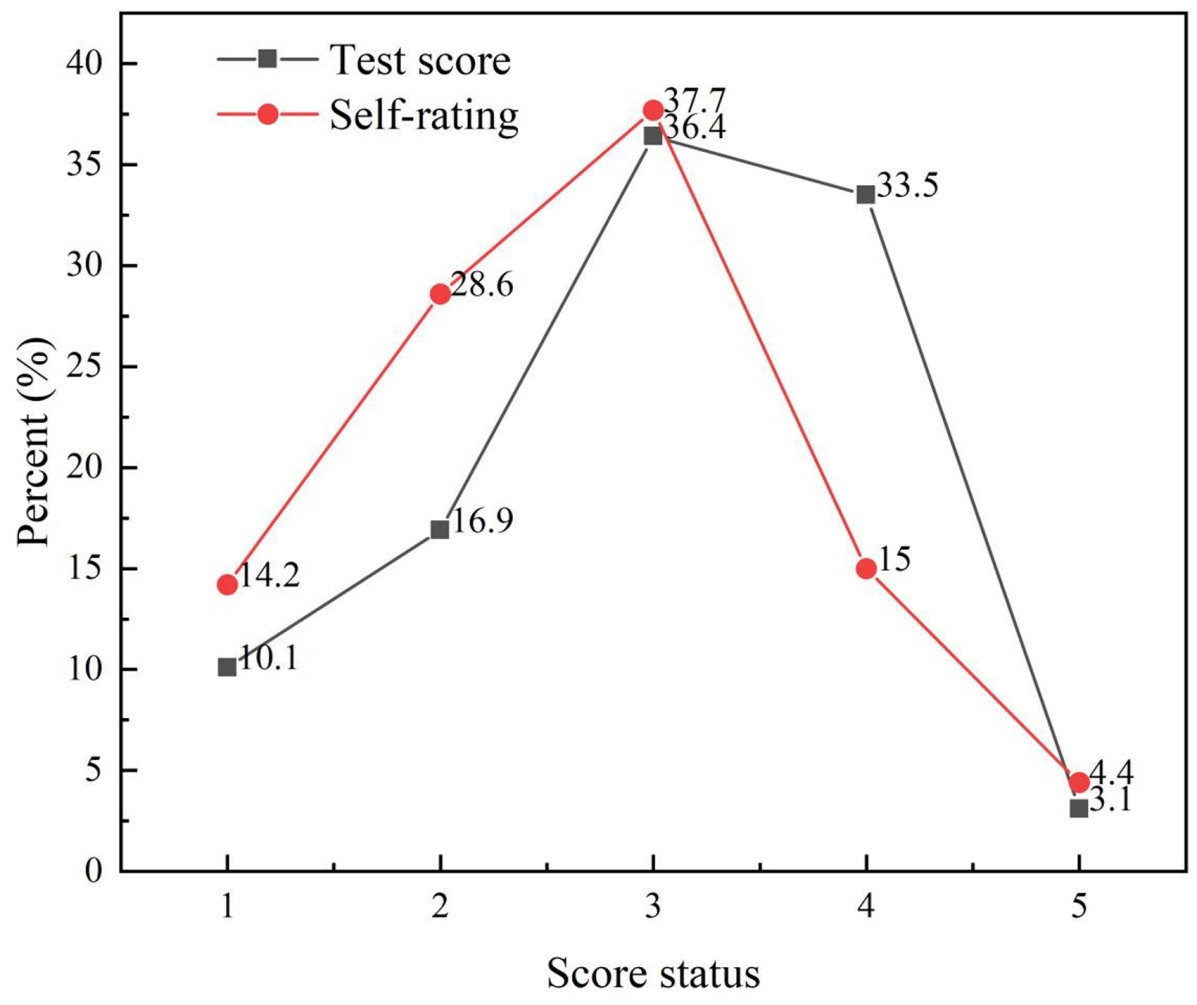
Difference between self-assessment scores and test scores of university students’ knowledge of waste classification.

**Figure 3:**
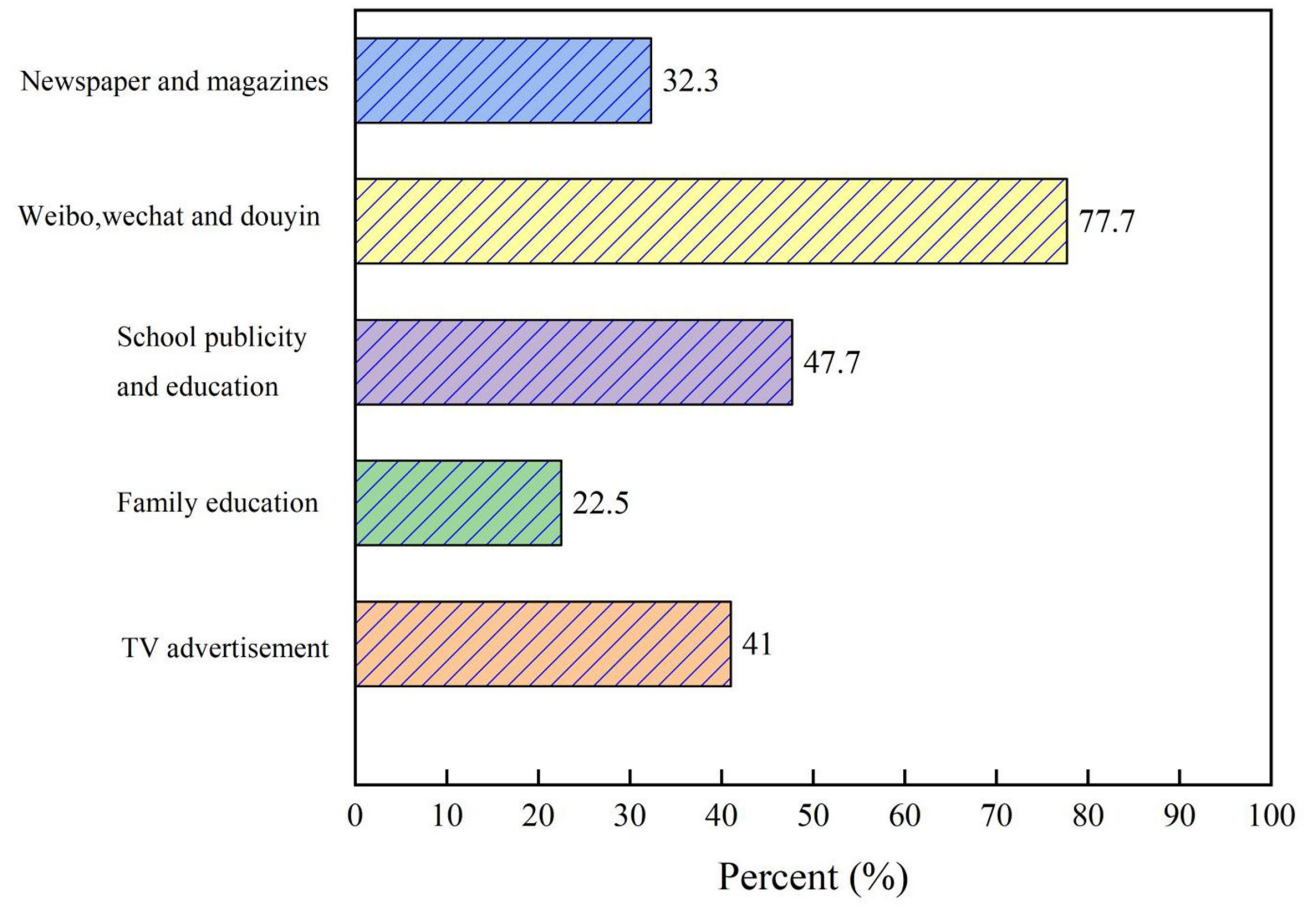
Main sources of college students’ knowledge of waste separation.

In the knowledge section of the survey, the average score of 1,282 university students regarding waste classification knowledge was 5.56 ± 2.05, with a score percentage of 55.64%, as shown in Table 1. The distribution of participants with completely correct, partially correct, and incorrect choices is illustrated in Figure 1. Table 2 displays the top three items with the highest frequencies of correct and incorrect responses. Additionally, by considering the supplementary self-assessment questions regarding waste classification knowledge for university students, it can be observed that there is a certain gap between self-evaluation and actual mastery (Figure 2). This observation reflects that some university students overestimated their proficiency in waste classification knowledge, as shown in Figure 3.

**Table 2:** The three items with the highest correct and incorrect rates in the knowledge section of university students’ waste classification.

	Number (%)	Mean ± SD
Highest number of all-correct		
1. Waste batteries are harmful garbage	84.3	0.84 ± 0.363
2. Spoiled sausages belong to kitchen waste	73.6	0.74 ± 0.441
3. Glass bottles are recyclable	72.5	0.73 ± 0.446
Highest number of false		
9. Chewing gum belongs to other garbage	28.2	0.28 ± 0.450
10. The leaves on the ground belong to kitchen waste	26.5	0.27 ± 0.442
8. Dust belongs to other garbage	34.1	0.35 ± 0.492

The results of the survey on the sources of knowledge show that the main sources are “WeChat, Weibo and short videos” (77.7%), “school propaganda” (47.7%), “TV advertisements” (41%), “newspapers and magazines” (32.3%), and “family education” (22.5%), as shown in Figure 3. To a certain extent, this reflects the neglect of waste classification education in schools and the failure of the main channel of education to fulfill its role.

### 3.3 Attitudes of university students toward waste separation

**Figure 4:**
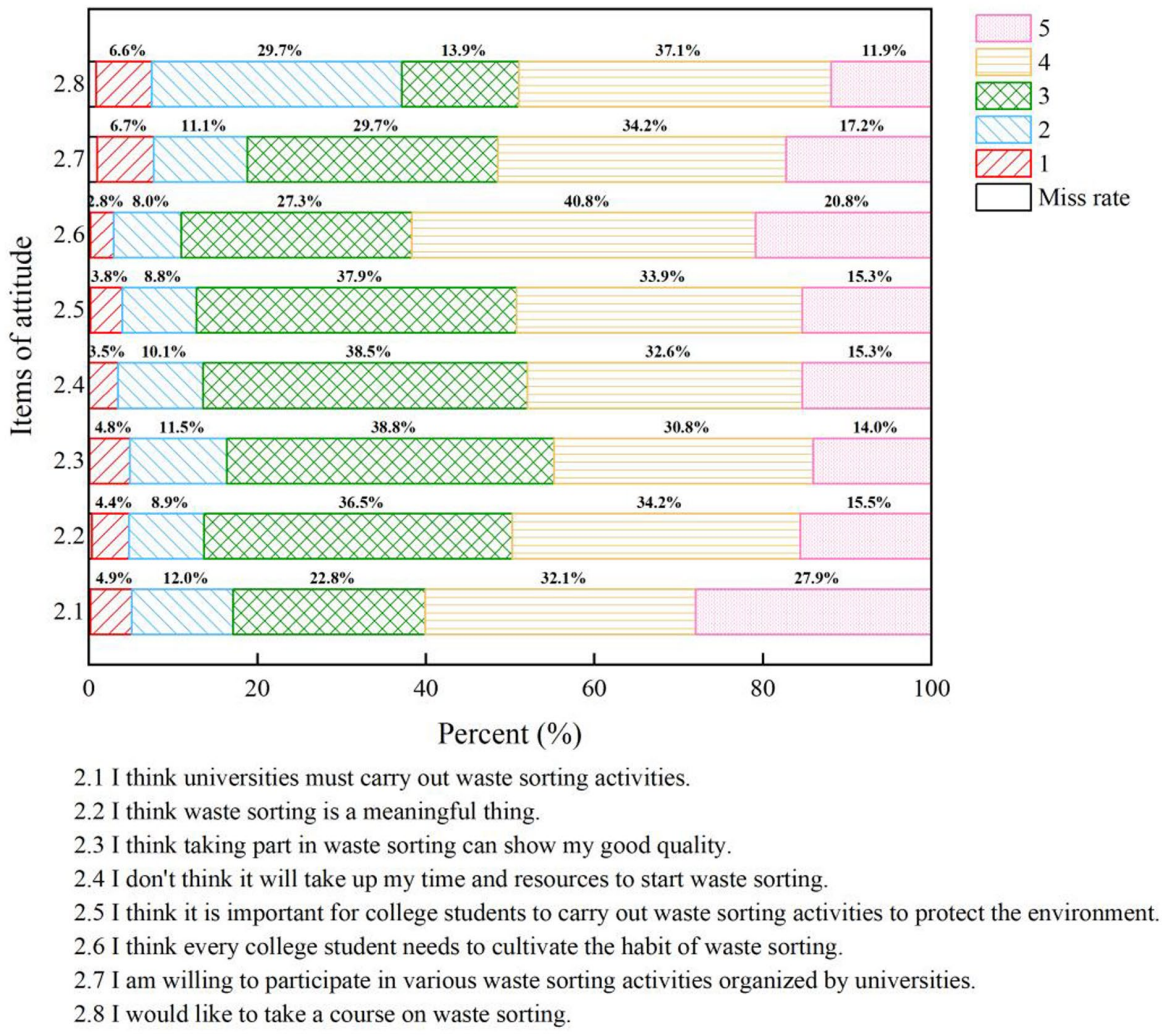
Assessment of university students’ attitudes toward waste separation (1–5 attitudes from weak to strong, the higher the value, the higher the level of recognition or importance).

The total attitude score of 1,279 university students (with three missing values) toward waste classification is 27.67 ± 6.28, with a score percentage of 69.18%, as shown in Table 1. The percentages for the eight questions on the waste classification attitude scale are illustrated in Figure 4. The survey indicates that the overall attitude of university students toward waste classification is positive. Approximately 70% of the surveyed students recognize the importance and necessity of waste classification.

### 3.4 University students’ practice of waste separation

**Figure 5:**
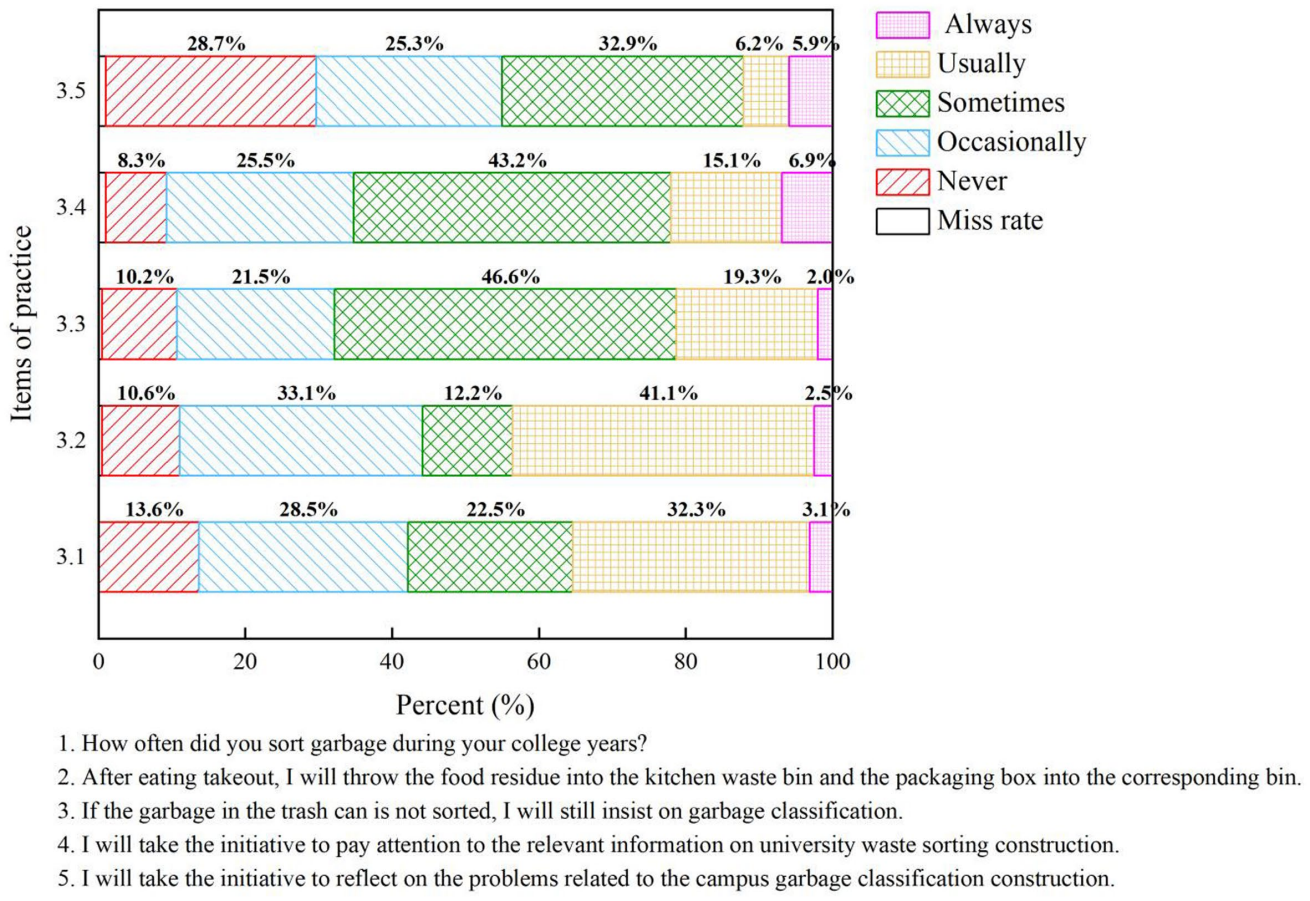
Assessment of university students’ waste separation practices.

According to participants’ reports, the practical scores of 1,281 university students (with one missing value) engaged in waste classification are 13.7 ± 3.97, with a score percentage of 54.8%, as shown in Table 1. The percentages for the five questions on the waste classification attitude scale are illustrated in Figure 5. The survey indicates that nearly half of the individuals have the motivation for waste classification, but only 454 individuals (35.4%) consistently adhere to it, and 174 students (13.6%) have never practiced waste classification (as shown in Question 3.1 in Figure 5). Additionally, 559 university students (43.6%) expressed that they would follow the classification standards when handling domestic waste in campus dormitories and academic buildings (as shown in Question 3.2 in Figure 5) and 273 students (21.3%) indicated that, even when the trash bin is in a mixed state, they would persist in waste classification (as shown in Question 3.3 in Figure 5).

In the practical survey of actively paying attention to waste classification information, the situation seems to be worse. Only 283 students (22%) stated that they actively pay attention to waste classification information. On the other hand, 106 students (8.3%) mentioned that they never pay attention to documents issued by the government or waste classification messages on media platforms. Additionally, 327 students (25.5%) expressed little interest (as shown in Question 3.4 in Figure 5).

In the practical survey of actively addressing waste classification issues, 368 individuals (28.7%) chose “never reflect the problem,” 324 individuals (25.3%) chose “rarely reflect the problem,” 422 individuals (32.9%) chose “occasionally reflect the problem,” 80 individuals (6.2%) chose “frequently reflect the problem,” and 76 individuals (5.9%) chose “always reflect the problem.” Additionally, 11 students chose other options and provided explanations in the blank space, accounting for 1% (as shown in Question 3.5 in Figure 5).

### 3.5 Factors influencing college students’ waste separation assessment scores

The total waste classification KAP score for 1,278 university students (with 4 missing values) is 46.93 ± 9.93, with a score percentage of 62.57% (Table 1). A one-way ANOVA, Pearson’s correlation analysis, and multiple linear stepwise regression analyzes were conducted to explore the factors affecting the scores. Seven demographic variables were set as independent variables, and scores for each section and KAP were set as dependent variables. The one-way ANOVA showed that college, grade, gender, major, specialization, highest education in the family, and family economic status affected the KAP scores of college students’ waste sorting behavior, as shown in Table 1. The results of the multiple linear stepwise regression analyzes are shown in Table 3. The variables affecting the total KAP score are familiarity with knowledge of waste classification (*β* = 0.545), gender (*β* = −0.133), major (*β* = −0.057), and political affiliation (*β* = −0.048). Gender was the most influential factor in the knowledge score. Knowledge familiarity with waste classification had a 52.1% influence on the attitude dimension. In the practice dimension, college students’ familiarity with waste classification knowledge (*β* = 0.38), gender (*β* = 0.16), grade level (*β* = −0.075), and highest education in the family (*β* = 0.71) were significantly correlated with the practice scores. The results of Pearson’s correlation analysis between the component scores and KAP scores are shown in Table 4.

**Table 3:** Multiple linear stepwise regression analyzes of KAP for waste separation among university students (*n* = 1,282).

	Variables	*R*	*R*^2^	Adjusted *R*^2^	B	Std. error	β	*t*	*F*	*p*-value
Knowledge score	Familiarity with garbage classification knowledge	0.311	0.097	0.096	0.614	0.052	0.311	11.705	137.015	0
	College	0.141	0.02	0.019	0.082	0.016	0.141	5.106	26.066	0
	Gender	0.130	0.017	0.016	0.537	0.115	0.13	4.68	21.899	0
	Grade	0.094	0.009	0.008	−0.153	0.045	−0.094	−3.375	11.393	0.001
Attitude score	Familiarity with garbage classification knowledge	0.534	0.285	0.285	3.231	0.143	0.534	22.578	509.78	0
	Grade	0.155	0.024	0.023	−0.772	0.138	−0.155	−5.604	31.403	0
	Family highest education	0.059	0.004	0.003	−0.413	0.195	−0.059	−2.122	4.501	0.034
	College	0.150	0.023	0.022	0.268	0.049	0.15	5.432	29.508	0
	Gender	0.102	0.01	0.01	1.2	0.353	0.102	3.679	13.539	0
Practice score	Familiarity with garbage classification knowledge	0.407	0.166	0.165	1.558	0.098	0.407	15.953	254.496	0
	Gender	0.202	0.041	0.04	1.625	0.22	0.202	7.39	54.61	0
	Grade	0.117	0.014	0.013	−0.367	0.087	−0.117	−4.197	17.618	0
	Family highest education	0.111	0.012	0.012	0.489	0.122	0.111	3.993	15.944	0
Total score	Familiarity with garbage classification knowledge	0.567	0.321	0.320	5.421	0.221	0.567	24.562	603.313	0.000
	Gender	0.173	0.030	0.029	3.478	0.554	0.173	6.281	39.456	0.000
	Grade	0.163	0.027	0.026	−1.287	0.218	−0.163	−5.913	34.965	0.000
	College	0.163	0.027	0.026	0.462	0.078	0.163	5.917	35.011	0.000
	Profession	0.071	0.005	0.004	0.402	0.158	0.071	2.540	6.452	0.011

**Table 4:** KAP Pearson’s correlation coefficient analysis of university students’ waste classification.

		College	Gender	Grade	Political status	Profession	Family highest education	Family economic situation	Familiarity with garbage classification knowledge
Knowledge score	r	0.141	0.130	−0.094−	0.023	−0.004	−0.017	0.004	0.311
	p	0	0	0.001	0.407	0.879	0.54	0.889	0
Attitude score	R	0.150	0.102	−0.155	0.021	0.070	−0.059	0.097	0.534
	P	0	0	0	0.459	0.012	0.034	0.001	0
Practice score	R	0.098	0.202	−0.117	−0.01	0.068	0.111	0.132	0.407
	P	0	0	0	0.713	0.015	0	0	0
Total score	R	0.163	0.173	−0.163	0.004	0.071	0.003	0.115	0.567
	P	0	0	0	0.88	0.011	0.916	0	0

### 3.6 Education and management of university students on waste separation in universities

**Figure 6:**
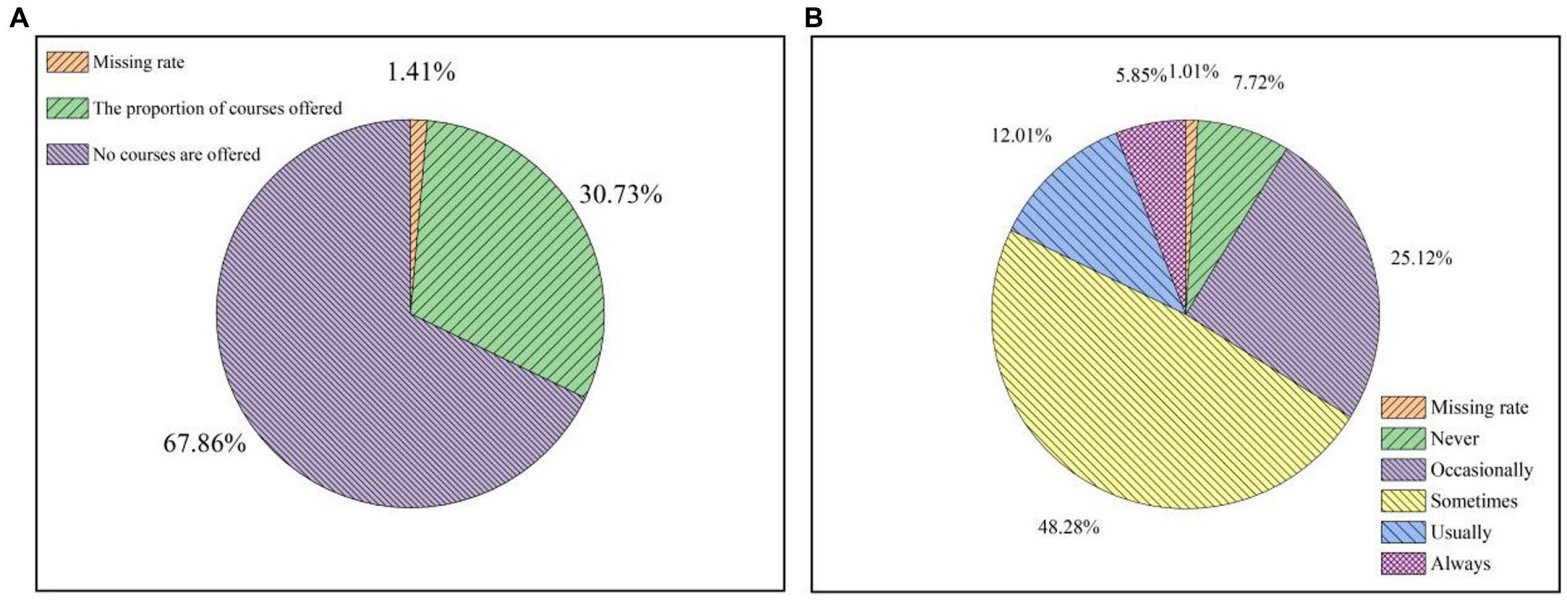
A survey on the education status of university students in colleges and universities **(A)** on the left asks whether relevant courses are offered; **(B)** on the right asks whether the teachers in the courses are lecturers on knowledge related to waste classification.

The data show that, among the school faculties of the research respondents, only 394 of them are in colleges that offer public courses about waste classification, accounting for only 30.73%, as shown in Figure 6A. In terms of course content, the interviewed college students said that it is not common for college teachers to teach garbage classification knowledge in class. The statistical results show that the percentages of never, occasionally, sometimes, often and always are 7.72, 25.12, 48.28, 12.01 and 5.85%, as shown in Figure 6B. 25.12, 48.28, 12.01, and 5.85%, as shown in Figure 6B. The survey shows that Chinese universities do not educate students sufficiently about waste separation and need to improve their curriculum.

**Figure 7:**
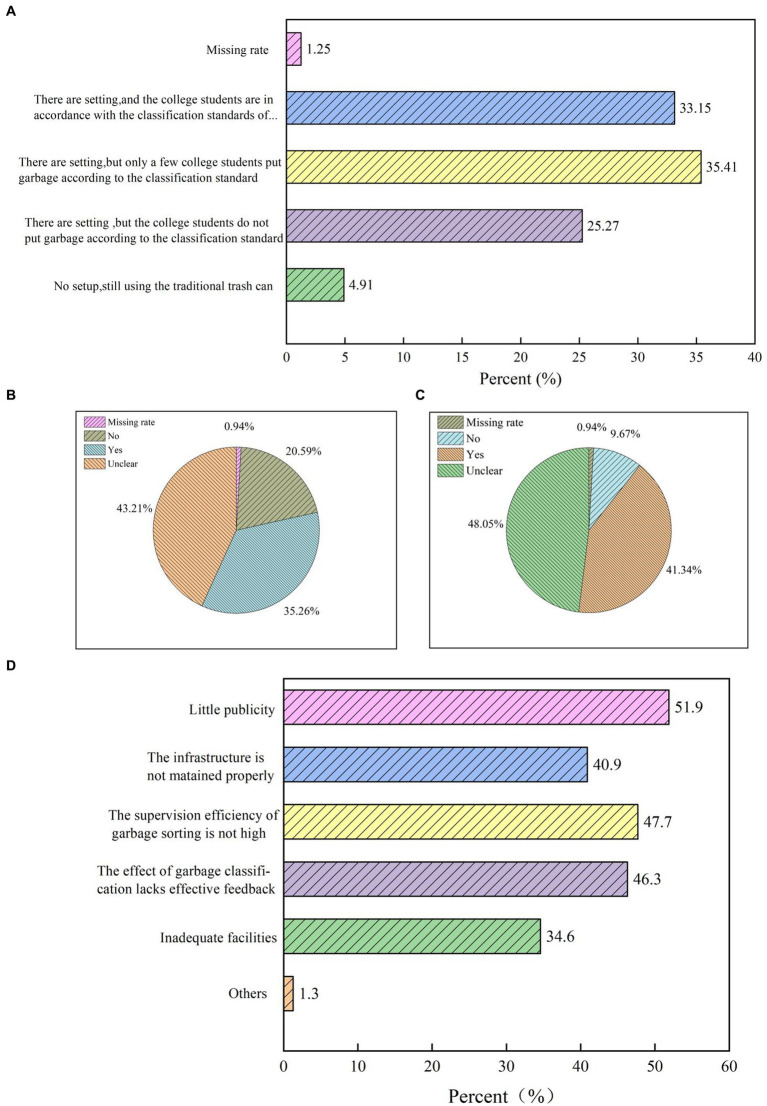
**(A)** Does your school have a special garbage sorting bin, and how is it being implemented? **(B)** Does your campus have a designated garbage sorting instructor? **(C)** Is there a regular inspection of the garbage sorting work on your campus? **(D)** What do you think is the problem with the garbage sorting work on your campus?

We included four questions on the issue of waste separation management in schools. As shown in Figures 7A–D, on the question of whether the school has set up special waste separation bins and their implementation, only 425 people said, “Yes, and students put out the waste by the standard of waste separation,” accounting for 33.15%. On the issue of the supervision of waste classification on campus, 554 people, or 43.21%, expressed uncertainty about whether there were special waste classification guides in schools and 616 people, or 48.05%, expressed uncertainty about whether the schools had arranged for special persons to check the situation of waste classification. In addition, regarding the issue of waste separation in schools, 665 people, or 51.9%, believed that the publicity on waste separation in schools was not strong and that the educational role had not been brought into play.

## 4 Discussion

As an important force for social development, the KAP status of university students on waste separation impact the progress and effectiveness of ecological civilization construction in China. Previous studies have shown significant differences in the implementation of waste separation among college students. It is necessary to assess the KAP levels of these university students to better optimize future education and management programs for university students’ waste separation. Therefore, to achieve this, we developed a comprehensive KAP questionnaire for waste sorting assessment, which encompasses several question types and supplementary questions to delve into relevant details. Our research team strictly adhered to the guidelines for questionnaire development, ensuring the scientific validity of the questionnaire. Rigorous testing for reliability and validity yielded positive results. This study represents the first exploration of the KAP levels regarding waste classification among university students. It sheds light on the KAP status, sources of knowledge, and influencing factors related to waste classification among college students in Beijing. This study also builds upon prior research by highlighting differences in the KAP levels of waste sorting among college students based on different majors, grades, genders, family educational backgrounds, and family income status. The findings also highlight that the current college students lack sufficient knowledge about waste sorting, exhibit a limited motivation to practice it, and suggest that the school education could play a potential role in improving the current status of waste sorting among college students.

The key findings of this survey on the KAP regarding waste separation among college students in Beijing, China can be summarized as follows: (1) The KAP level is deemed average and requires improvement. Although college students exhibit a positive attitude toward waste separation, they possess insufficient knowledge, demonstrate weak practical performance, and are prone to making mistakes during the implementation process. (2) The KAP level of waste separation varies significantly among college students from different majors, with the highest rankings observed in literature majors. (3) A lack of knowledge is the main obstacle in the process of waste separation. (4) The main sources of knowledge about waste separation for college students are platforms such as WeChat, Weibo, and short videos, highlighting the failure of school education to fulfill its role in this regard. (5) Due to the lack of knowledge, scientific training, and school education on waste separation, college students do not adhere to proper waste separation practices. (6) College students’ performance falls short of the expected standard. (7) College students’ proficiency in waste separation is not as good as it should be. (8) Due to the absence of knowledge, scientific training, and specialized education in schools, the implementation of waste separation by college students is not satisfactory. (9) The practice of waste separation by college students is based on experience. (10) A majority of the faculties and departments in colleges and universities do not offer courses on waste separation, putting their supervision and management at a disadvantage.

What can be seen in the knowledge test of university students on waste classification is that the knowledge of university students on waste classification is not satisfactory, and the knowledge education needs to be strengthened. The results of the research show that nearly half of the students do not yet know the standards of the four categories of waste classification: food waste, recyclable waste, hazardous waste, and other waste, and the average score of 1,282 college students on the knowledge of waste classification is only 5.56 (out of 10), which is consistent with the findings of the study of Ai et al. (43). In addition, we found that first-year university students performed significantly better than other years in terms of their knowledge of waste classification. This can be attributed to the fact that university students need to receive a civics course at this stage, aligning with the findings of Liu’s study (44). Furthermore, in terms of gender differences, female students (mean score of 5.87) exhibited greater familiarity with knowledge of waste classification compared to male students (mean score of 5.34). This finding aligns with the findings of Li’s study, which reflects that girls are more likely to be influenced by subjective norms, whereas boys are more likely to be influenced by perceptual, behavioral control when sorting waste (45). In addition, when examining the three items with the highest correct and incorrect rates of waste classification knowledge, it becomes evident that college students have a better understanding of recyclable waste and hazardous waste. Conversely, knowledge regarding food waste and other types of waste tends to be more easily ignored by college students. The educational process within schools should also work on these two categories of knowledge. This finding is also related to the results of the study of Li et al., which reflects that girls are more likely to be influenced by subjective norms, while boys are more likely to be influenced by perceptual behavioral control in the context of waste classification. This finding aligns with the results of the study of Peng et al. (46). In addition, we found that there is a discrepancy between the self-assessed scores and the actual test results of college students’ knowledge of waste classification, which indicates that college students generally overestimate their knowledge of waste classification. Combining the results of the knowledge and practice dimensions of university students’ waste sorting, we hypothesize that deficiency in knowledge regarding waste sorting among university students will impact their practice of waste sorting.

This study investigated the attitudinal dimension of university students’ knowledge of waste classification. The data showed that college students scored 69.18% in the attitude dimension of waste separation, which means that the majority of college students maintain a very positive attitude toward waste separation, a finding similar to that of Yuxuan and Kaiyang’s study (47). This study also found that both the knowledge and attitude dimensions exhibit a more significant predictive influence on the practice dimension, that is, the higher the knowledge and better the attitude toward waste separation, the more accurate their waste separation behaviors tend to be. This aligns with the findings of previous studies (48).

In terms of the practice dimension, only 444 people were proficient in implementing waste sorting behaviors, accounting for 35.4% of the total research population. Our findings reflect that college students’ proficiency in waste sorting shows a correlation with factors such as grade level, gender, parental education, and family economic status.

This study confirms that knowledge familiarity is one of the important factors for university students to carry out waste classification. In this survey, through multiple linear stepwise regression analyzes, we found that knowledge familiarity had a more significant effect on the total score and KAP. The survey shows that students majoring in humanities and social sciences exhibit a better understanding of waste classification, probably because of the inclusion of relevant courses in their academic curriculum (49). In this study, in addition to knowledge familiarity, factors such as gender, grade, major, and family income also had an impact on college students’ KAP scores on waste classification. This finding reveals the need to tailor the educational content and teaching methods to enhance college students’ practice of waste classification.

In the research aspect of the school teaching and management dimension, it is evident that the efforts in waste classification publicity and education within colleges and universities are still relatively inadequate. In particular, colleges and universities hardly teach how to classify garbage in class, and rarely involve explanation of waste classification knowledge in extracurricular activities, resulting in insufficient understanding of the importance of waste classification among college students. This lack of knowledge about waste classification affects their motivation to participate in waste classification practices. In addition, colleges and universities generally lack the necessary service and management facilities for waste classification. The hardware conditions for waste classification are insufficient. The investment in waste classification in colleges and universities is not enough. Additionally, there is a lack of corresponding system construction for effective waste management. In terms of suggestions, first, based on universally setting up recycling bins in colleges and universities, we should study and improve the appropriateness of on-campus waste classification facilities. This study includes improvements in terms of logo design, publicity content, and the establishment of classification points to reinforce the students’ rubbish classification behaviors. Second, there is a need to deepen education on waste classification in colleges and universities so that students not only know what to do but also comprehend the underlying reasons. They must understand the principles of rubbish classification, its methodologies, and the ultimate destinations of the classified waste. This education process can garner support from professional social organizations. Third, the stakeholders who participate in the process are college students, being not only the main contributors to campus waste classification but also the potential volunteers, that can play a crucial role. Only through active participation and adherence can they become faithful practitioners of waste classification.

Indeed, for optimizing KAP scores for students’ waste sorting, we can apply Donella Meadows’ concept of creating a deep leverage point. This entails reshaping students’ fundamental values, life goals, and worldviews (50, 51). Reconstruct, reconnect, and rethink human–nature interactions by conveying conscientious and sustainable values for our time to students through science, art, literature, and life experiences (52, 53).

There are some limitations in this study. First, concerning the sample size, the formal questionnaire in this study collected 1,282 valid responses, meeting the basic requirements for structural equation modeling analysis. However, the sample source of the questionnaire is mainly from colleges and universities in the Beijing area, necessitating caution regarding the representativeness and comprehensiveness of the questionnaire. Second, due to the complexity of the question types and content, the questionnaire did not incorporate reverse scoring. In addition, during the development of the questionnaire, although 10 experts gave consistent opinions, it is noteworthy that they all belonged to the same school. Finally, some of the respondents provided somewhat invalid answers, potentially introducing selection bias into the study.

## 5 Conclusion

According to the results of this study, the overall KAP score of waste separation among Chinese college students is barely acceptable. The interviewed college students show a positive attitude and willingness to engage in waste separation. However, the state of both knowledge and practical implementation needs to be changed. Uncertainty about the knowledge of waste classification is the predominant factor influencing individual-level waste separation practices. Consideration should be given to strengthening the education and management of waste separation among university students, with a focus on the cultivation of eco-surplus culture and guiding students to establish correct ecological values. We also suggest that, in the process of strengthening university students’ education on waste separation, it is necessary to take into account the actual situation of the target students and provide targeted and classified guidance, taking into account factors such as gender, grade, major, and family situation. In addition, the KAP assessment tool for waste separation needs to be revised for practical use.

## Data availability statement

The datasets presented in this study can be found in online repositories. The names of the repository/repositories and accession number(s) can be found at: https://osf.io/4kxf5/?view_only=2c4d75e5553548588fdb1bdf545c3570.

## Ethics statement

Ethical approval was not required for the study involving humans in accordance with the local legislation and institutional requirements. The studies were conducted in accordance with the local legislation and institutional requirements. Written informed consent to participate in this study was not required from the participants in accordance with the national legislation and the institutional requirements.

## Author contributions

SL: Data curation, Writing – original draft, Writing – review & editing. XL: Investigation, Resources, Writing – review & editing. YL: Formal analysis, Visualization, Writing – review & editing. DY: Project administration, Software, Writing – review & editing. FL: Supervision, Writing – review & editing. JY: Conceptualization, Funding acquisition, Resources, Supervision, Writing – review & editing.
